# Association Between Single-Nucleotide Polymorphism and Trastuzumab Deruxtecan-Induced Interstitial Lung Disease in Breast Cancer Using the Japonica Array NEO

**DOI:** 10.3390/cancers18060927

**Published:** 2026-03-12

**Authors:** Saori Fujiwara, Nao Saito, Mio Yasukawa, Akira Narita, Mika Sakurai-Yageta, Shinya Sato, Toshinari Yamashita, Daisuke Hoshino

**Affiliations:** 1 Department of Breast Surgery, Kanagawa Cancer Center, Yokohama 241-8515, Japan; s-fujiwara@kcch.jp (S.F.); yasukawa.as40n@kanagawa-pho.jp (M.Y.); yamashita.0610h@kanagawa-pho.jp (T.Y.); 2 Cancer Biology Division, Kanagawa Cancer Center Research Institute, Yokohama 241-8515, Japan; nasaito22@gmail.com; 3 Biospecimen Center, Kanagawa Cancer Center, Yokohama 241-8515, Japan; 4 Tohoku Medical Megabank Organization, Tohoku University, Sendai 980-8573, Japan; akira.narita.b6@tohoku.ac.jp (A.N.); mika.sakurai.a4@tohoku.ac.jp (M.S.-Y.); 5 Molecular Pathology and Genetics Division, Kanagawa Cancer Center Research Institute, Yokohama 241-8515, Japan; sato.0f80j@kanagawa-pho.jp; 6 Morphological Analysis Laboratory, Kanagawa Cancer Center Research Institute, Yokohama 241-8515, Japan

**Keywords:** trastuzumab deruxtecan, interstitial lung disease, single-nucleotide polymorphism

## Abstract

**Background/Objectives**: Trastuzumab deruxtecan (T-DXd) is an effective antibody–drug conjugate for human epidermal growth factor receptor 2-expressing solid tumors; however, interstitial lung disease (ILD) remains a clinically significant adverse event. Although both clinical characteristics and genetic susceptibility have been implicated in drug-induced ILD, the factors specifically associated with T-DXd-induced ILD remain unclear. This study aimed to evaluate clinical and genetic factors associated with the development of ILD in patients treated with T-DXd. **Methods:** We retrospectively analyzed the clinical data of 54 patients treated with T-DXd. The baseline clinical and treatment-related characteristics of patients with ILD and those without were compared. Genome-wide single-nucleotide polymorphism (SNP) analyses with genotype imputation and targeted candidate SNP analyses were performed to evaluate genetic susceptibility. **Results:** ILD occurred in 15 patients (27.8%), with a median time to onset of 215 days (range, 48–1187). None of the baseline clinical or treatment-related factors were significantly associated with ILD development. Genome-wide analyses did not reveal any significant SNPs after correcting for multiple testing. Contrarily, a targeted analysis focusing on SNPs previously associated with drug-induced ILD identified one variant rs12625311 that was significantly associated with ILD in this cohort. **Conclusions:** In this exploratory study, baseline clinical characteristics alone were insufficient to discriminate the risk of ILD among patients treated with T-DXd. Although no high-penetrance genetic variants were identified, candidate-based genetic analyses suggested that host genetic factors may contribute to ILD susceptibility. Integrating genetic information with clinical assessment may help refine the risk stratification for T-DXd-induced ILD in future studies.

## Simple Summary

Trastuzumab deruxtecan is an effective treatment agent for a wide range of cancers, including those with low human epidermal growth factor receptor 2 expression. However, it can cause interstitial lung disease, a side effect that may sometimes be life-threatening, often requiring treatment interruption. Currently, the prediction of patients at a higher risk of developing this complication is difficult. In this study, we examined whether patient background factors, such as age, kidney function, or treatment dose could explain the risk of developing lung disease. Additionally, we explored whether inherited genetic differences might play a role. Our data did not demonstrate that common clinical factors alone were not sufficient to identify high-risk patients. However, our genetic analysis suggested that certain genetic variations may influence individual susceptibility. These findings indicate that trastuzumab deruxtecan-induced lung toxicity is complex and may depend on both treatment effects and patient-specific factors, supporting future research on more personalized risk assessments.

## 1. Introduction

Trastuzumab deruxtecan (T-DXd) is a human epidermal growth factor receptor 2 (HER2)-directed antibody–drug conjugate that demonstrates efficacy across multiple HER2-expressing malignancies, including HER2-positive and HER2-low diseases [1,2,3].

Despite its clinical efficacy, interstitial lung disease (ILD) has emerged as one of the most significant adverse events associated with T-DXd therapy. The incidence of T-DXd-induced ILD has been reported to range from approximately 11% to 16%, with most cases being low grade; however, severe and fatal cases have also been documented [4,5,6,7]. Drug-induced ILD is widely recognized as a potentially life-threatening toxicity that may rapidly and substantially affect morbidity and mortality, often necessitating the discontinuation of otherwise effective anticancer therapies [8,9].

Importantly, a consistently higher incidence of drug-induced ILD has been reported in Japanese than in non-Japanese patients [8,10,11]. In the pooled analyses of T-DXd monotherapy studies, the incidence of ILD in Japanese patients was approximately two-fold higher than that in non-Japanese patients, and Japanese ancestry was identified as an independent risk factor in multivariate analyses [3,5,12]. Although differences in surveillance practices and clinical management may partly influence the reported incidence [8,10,11], these factors do not fully explain the reproducible elevation in ILD risk.

Clinical characteristics such as age, pre-existing lung disease, smoking history, and cumulative exposure to anticancer agents have been reported to be associated with drug-induced ILD [5,8,9,13]. A comprehensive review highlighted that drug-induced ILD represents a heterogeneous clinical entity with non-specific radiological and pathological features, reflecting a complex interplay between drug-related toxicity and host-related factors rather than a single causal mechanism [14]. Accordingly, clinical risk factors alone do not sufficiently account for interindividual variability in susceptibility.

Modest pharmacokinetic differences between Japanese and non-Japanese patients have been reported, with slightly higher systemic exposure in Japanese patients [15]. However, their clinical relevance to ILD risk remains uncertain.

In addition to clinical and pharmacokinetic influences, host genetic susceptibility has been proposed as a contributing factor in drug-induced ILD. However, the evidence linking germline single-nucleotide polymorphisms (SNPs) to anticancer drug-induced ILD remains limited. To date, only one comprehensive study has systematically evaluated germline variants associated with drug-induced ILD in Japanese patients with cancer using whole-genome sequencing, with the reported associations having not yet been widely replicated [16]. Associations between specific human leukocyte antigen alleles and drug-induced ILD have been reported in selected clinical settings, such as gemcitabine plus erlotinib-induced ILD in Japanese patients, suggesting a potential role for immune-related genetic predisposition [17]. Moreover, SNPs associated with ILD have been described in non-drug-induced contexts, such as rheumatoid arthritis-associated ILD, further support the concept that the host genetic background may influence susceptibility to interstitial lung injury, although their relevance to anticancer drug-induced ILD remains unclear [18].

Currently, the mechanisms underlying T-DXd-induced ILD have not been fully elucidated. Pre-clinical and translational studies suggest that lung injury may not be solely explained by HER2-dependent target engagement, raising the possibility that host-related factors contribute to susceptibility [7,19,20]. Nevertheless, the factors underlying individual susceptibility to T-DXd-induced ILD remain incompletely understood, particularly in Japanese patients at increased risk.

In this study, we investigated the clinical and genetic factors associated with T-DXd-induced ILD in Japanese patients with metastatic or recurrent breast cancer. To explore genetic susceptibility, we used a Japanese population-optimized SNP array. We also examined previously reported ILD-related variants in Japanese patients to better characterize susceptibility in a high-risk population.

## 2. Materials and Methods

### 2.1. Patients

This was a retrospective cohort study; the study protocols were approved by the Ethics Committee of Kanagawa Cancer Center (approval number # 28KEN42, 2023EKI64). The study adhered to the ethical principles of the Declaration of Helsinki. Written informed consent for participation in the study, including genetic analysis, was obtained from patients before blood samples were collected.

Patients with metastatic or recurrent breast cancer who initiated treatment with T-DXd at Kanagawa Cancer Center between November 2017 and February 2025 were screened for eligibility. For patients who received T-DXd as part of a clinical trial, only those for whom permission to use the clinical data for research purposes were granted by the study sponsors were included in the analysis. Patients with available clinical data and blood samples were included in the analysis. Patients were followed up until 31 August 2025. Patients who did not develop ILD were included in the analysis only if they had received at least three cycles of T-DXd treatment.

All patients included in this study were Japanese and their ethnicity was confirmed from medical records. HER2 expression was assessed by immunohistochemistry (IHC) and recorded as an IHC score of 1+, 2+, or 3+.

### 2.2. Assessment of T-DXd-Induced ILD

T-DXd-related ILD was defined as newly developed interstitial lung abnormalities on chest computed tomography (CT) after treatment initiation, irrespective of symptoms.

Radiologic findings considered consistent with ILD included ground-glass opacities, organizing pneumonia-pattern consolidation, reticular abnormalities, and diffuse alveolar damage-type changes. Nodular or lymphangitic patterns suggestive of tumor progression were not classified as ILD.

Alternative causes, including infection (e.g., cytomegalovirus or COVID-19), tumor progression, and radiation-induced lung injury were excluded based on clinical course, treatment history, available laboratory data, and imaging features. Microbiological testing was performed when clinically indicated. Serum KL-6 and SP-D levels were measured as supportive biomarkers but were not used as definitive diagnostic criteria due to their limited specificity.

Final diagnosis was determined by integrating radiologic findings with clinical assessment. ILD was diagnosed by at least one pulmonologist and multiple oncologists in conjunction with radiology reports.

Chest CT was performed at routine intervals for disease monitoring. Additional imaging was obtained when clinically indicated.

ILD severity was graded according to the Common Terminology Criteria for Adverse Events (CTCAE) version 1.1.

### 2.3. Clinical Data Collection

Clinical data were retrospectively collected from medical records and included patient demographics, disease characteristics, treatment history, and ILD occurrence during T-DXd therapy.

### 2.4. Genomic DNA Preparation

Blood samples were collected at any time point after cancer diagnosis, either before or during T-DXd treatment. Germline variants were analyzed to assess host genetic factors. As germline variants are generally stable and not expected to be influenced by treatment exposure, sampling timing was unlikely to affect the analyses.

Peripheral blood samples were collected using 5 mL EDTA-2Na blood collection tubes after obtaining written informed consent from patients. Plasma was separated from the collected blood by centrifugation, and the remaining whole blood sample was used for DNA extraction after adding 2 mL of phosphate-buffered saline. Genomic DNA was extracted from plasma-depleted whole blood using a fully automated DNA extraction system, QuickGene-Auto 240 L (Kurashiki Boseki Co., Ltd., Osaka, Japan).

### 2.5. Genotyping with the Japonica Array NEO

Genotyping was performed using the Japonica array NEO, developed as a useful tool for genotyping Japanese populations [21]. Genotyping assays were conducted according to the manufacturer’s instructions (Axiom™ 2.0 Assay 96-Array Format Manual Workflow (Thermo Fisher Scientific, Waltham, MA, USA)). Briefly, target DNA was enzymatically amplified and fragmentated. After confirming concentration and fragment length using NanoDrop (Thermo Fisher Scientific, Waltham, MA, USA) and 4% agarose gel electrophoresis, hybridization, ligation, and scanning were performed using the semi-automated GeneTitan™ Multi-Channel Instrument (Thermo Fisher Scientific). For quality control (QC), following the Axiom™ Genotyping Solution Data Analysis Guide, the Axiom™ Analysis Suite software 5.2 was used to analyze dish QC, sample QC call rate, and plate pass rate using the Axiom platform’s control markers (approximately 19,000).

### 2.6. Genotype Imputation and Genome-Wide Association Study

Genotyping data were subjected to SNP QC checks and genotyping imputation at the Tohoku Medical Megabank Organization GWAS Center. To ensure data quality, we performed comprehensive QC at SNP, sample, and plate levels, utilizing all available markers across each plate. After filtering out variant sites with low call rates, low minor allele frequency (MAF), or those showing substantial deviation from the Hardy–Weinberg equilibrium, SHAPEIT2 [22] and IMPUTE2 [23] were used to conduct pre-phasing and genotype imputation, respectively. Imputation accuracy was evaluated using the squared correlation (r^2^) with leave-one-out SNP masking methods. Briefly, we executed the genotype imputation by masking an input SNP. The imputed SNP was compared with the masked SNP to obtain the r^2^, after which the average r^2^ in each MAF bin was calculated. Another metric, the information measure (INFO score) provided by IMPUTE2, was used to analyze the imputation quality for each marker, where a value in the range of 0–1 indicated uncertainty about the imputed genotype [21,24].

### 2.7. Statistical Analysis

Associations between baseline categorical variables and ILD development were evaluated using univariate Firth-penalized logistic regression, and the results are reported as odds ratios (ORs) with 95% confidence intervals (CIs). Continuous variables (age, creatinine clearance, body weight, and HER2 immunohistochemistry score) are summarized as medians with interquartile ranges (IQRs), and they were compared between ILD+ and ILD− patients using the Mann–Whitney U test.

## 3. Results

### 3.1. Patient Characteristics and Clinical Course of ILD

Table 1 presents the baseline characteristics of patients treated with T-DXd according to ILD development in the study population. Continuous variables are shown as a median (range), and categorical variables as a number (%). Eastern Clinical Oncology Group (ECOG) performance status was dichotomized as 0 vs. ≥1. Creatinine clearance (CrCl) was calculated using the Cockcroft–Gault formula. HER2 equivocal cases were categorized as HER2-negative.

A total of 54 patients treated with T-DXd were included in the analysis. ILD developed in 15 patients (27.8%), whereas 39 (72.2%) did not develop ILD during the observation period (Table 1). All ILD events occurred during ongoing T-DXd treatment. No patients were receiving concomitant systemic anticancer therapy at the time of ILD onset.

Among the patients who developed ILD, the median time from T-DXd initiation to ILD onset was 215 days (range, 48–1187 days). Six of the 15 ILD cases occurred more than 12 months after treatment initiation. According to the CTCAE criteria, 11 and 4 patients experienced Grade 1 and 2 ILD, respectively. No Grade ≥3 events were observed, and consequently no fatal ILD events occurred during the study period. Corticosteroid therapy was administered to two patients for ILD management.

Patients who did not develop ILD were followed up for a median of 273 days (range, 57–2557 days). During follow-up, treatment discontinuation in the non-ILD group occurred primarily due to disease progression (30 of 39 patients), whereas one patient discontinued treatment due to cirrhosis. By the end of follow-up, eight patients remained on T-DXd treatment.

The baseline patient characteristics stratified by ILD status are summarized in Table 1. Overall, the distributions of demographic characteristics, performance status, comorbid conditions, tumor-related factors, and treatment-related variables were broadly comparable between patients with and without ILD.

### 3.2. Clinical Factors Associated with T-DXd-Induced ILD

Table 2 presents univariate analyses for factors associated with ILD development. ORs with 95% CIs were estimated using Firth penalized logistic regression to reduce small-sample bias.

Based on prior reports suggesting that clinical characteristics may influence susceptibility to T-DXd-induced ILD, we explored the association between pre-specified baseline factors and ILD development in this cohort. These analyses focused on clinically relevant variables, including patient demographics, pulmonary and smoking histories, tumor characteristics, and treatment-related factors.

Univariate Firth-penalized logistic regression analyses were performed to evaluate the associations between baseline categorical variables and ILD development (Table 2). No factors demonstrated a statistically significant association with the occurrence of ILD in this exploratory analysis. Specifically, older age (≥65 years), ECOG performance status ≥1, presence of lung comorbidities, smoking history, lung or pleural metastasis, impaired renal function (creatinine clearance < 90 mL/min), high baseline T-DXd dose (≥6.4 mg/kg), greater number of prior lines of therapy (≥3 vs. ≤2), estrogen receptor status, HER2 status (with equivocal categorized as negative), and HER2 IHC category (≥2+ vs. ≤1+) were not significantly associated with ILD development.

In addition to the categorical analyses, continuous variables were evaluated to further explore their potential associations with ILD development. Median age did not significantly differ between ILD+ and ILD− patients (63.0 [IQR 52.0–68.5] vs. 58.0 [48.5–65.0] years, respectively; Mann–Whitney U test, *p* = 0.254). Similarly, baseline renal function assessed by CrCl showed no significant difference between the two groups (74.4 [54.2–86.8] vs. 85.5 [71.0–99.4] mL/min, respectively; *p* = 0.189). Body weight at treatment initiation was also comparable between patients with and without ILD (46.1 [41.7–57.8] vs. 51.3 [43.8–61.0] kg, respectively; *p* = 0.451).

HER2 expression assessed as a continuous IHC score (1+, 2+, or 3+) did not differ significantly between ILD-positive and ILD-negative patients (median IHC score 2.0 [IQR 1.0–3.0] vs. 2.0 [1.0–2.0], respectively; *p* = 0.238). These findings were consistent with the results of the categorical analyses and did not identify any continuous baseline variables that were significantly associated with ILD development.

Time-to-ILD was evaluated using univariate Cox proportional hazards models accounting for censoring; however, no factor showed a statistically significant association.

Overall, no baseline clinical or treatment-related factors were significantly associated with ILD development in this exploratory analysis.

### 3.3. Association Analysis of Previously Reported T-DXd-Induced SNPs

To further investigate the potential determinants of ILD risk beyond the clinical factors, we examined whether genetic variations were associated with ILD development in this cohort. All patients were confirmed to be Japanese ethnicity based on their medical records (Table 1). Given that Japanese ethnicity is a known risk factor for T-DXd-induced ILD, we focused on population-specific genetic susceptibility. We conducted a genome-wide association study (GWAS) using the Japonica Array NEO, which detects approximately 675,000 SNP probes. This array is specifically designed for the Japanese population by combining SNPs selected from Japanese whole-genome data and disease-associated SNPs, providing high coverage of the Japanese genomic background [21]. As a screening study, we conducted a case–control association study between 15 ILD+ and 39 ILD- (non-ILD) patients to identify specific single-nucleotide variants associated with T-DXd-induced ILD. However, no variants reached genome-wide significance (*p* < 5 × 10^−8^).

Consequently, we proceeded to the targeted evaluation of 42 loci previously reported to be associated with drug-induced ILD in Japanese patients with cancer [16]. Because this previous report primarily focused on other anti-cancer agents and did not include T-DXd, we examined whether these established variants were also relevant to T-DXd. By calculating allele frequencies at 21 of the 42 loci, one single-nucleotide variant, rs12625311, which is located within the intron regions of *TSHZ2* showed a nominal association with the development of T-DXd-induced ILD (*p* = 1.59 × 10^−2^, Table 3). Interestingly, the risk allele identified in our cohort was opposite to that reported in a previous study of other anti-cancer agents [16].

## 4. Discussion

In this study, we investigated genetic susceptibility to T-DXd-induced ILD in a Japanese breast cancer cohort using an ethnicity-specific SNP array. A genome-wide association analysis comparing patients with and without ILD did not identify any variants that reached genome-wide significance, even after genotype imputation expanded the coverage beyond the variants directly included in the array. However, targeted analyses of previously reported ILD-associated SNPs in the Japanese population revealed a significant association for one variant, although the direction of this association was opposite to that reported in a previous study [16]. These findings suggest that host genetic factors may modulate susceptibility to T-DXd-induced ILD, while also highlighting the complexity of genetic contributions to drug-induced lung disease.

The pathogenesis of T-DXd-induced ILD remains incompletely understood, and multiple non-mutually exclusive mechanisms have been proposed. Although HER2 is detectable in normal lung tissues at the RNA and protein levels, the incidence of HER2 antibody–drug conjugate-associated ILD does not correlate with target antigen expression or IHC intensity, suggesting that the pulmonary toxicity of HER2-directed antibody–drug conjugates cannot be explained solely by HER2 dependent target engagement [25]. To this end, off-target effects related to payload release and intracellular processing have been implicated. The cleavable linker of T-DXd is designed to be processed by lysosomal proteases, such as cathepsins, which are expressed not only in tumor cells, but also in immune and inflammatory cells, raising the possibility of bystander toxicity in pulmonary tissue [26].

Non-clinical studies have provided further support for these hypotheses. In a cynomolgus monkey model, repeated administration of T-DXd induced histopathological lung changes consistent with interstitial pneumonitis, whereas administration of the unconjugated payload did not reproduce these findings, suggesting that free deruxtecan alone was insufficient to induce lung injury [19]. Reviews of anticancer therapy-related ILD have emphasized that pulmonary toxicity is a multifactorial process involving epithelial injury, immune activation, and dysregulated repair responses, rather than a single causal pathway [7,20].

In the present study, none of the baseline clinical or treatment-related factors examined were significantly associated with the development of T-DXd-induced ILD. These findings should be interpreted in the context of previous reports suggesting that the clinical risk factors for drug-induced ILD, including advanced age, pre-existing lung disease, smoking history, renal impairment, and cumulative exposure to anticancer therapies, may vary across agents and patient populations.

With respect to treatment-related variables, baseline dose category (≤5.4 vs. ≥6.4 mg/kg) was not associated with ILD occurrence in our cohort. This observation is consistent with pooled analyses of T-DXd monotherapy studies, in which an increased risk of ILD was suggested at doses exceeding 6.4 mg/kg, whereas no clear risk difference was observed between the 5.4 and 6.4 mg/kg dose levels [5]. Direct pharmacokinetic measurements were not performed in this study; therefore, the potential impact of interindividual differences in drug exposure cannot be fully excluded.

With respect to renal function, baseline renal impairment was not associated with ILD development in our analysis, in which renal function was estimated using the Cockcroft–Gault formula. In a pooled analysis of T-DXd monotherapy studies, renal impairment was suggested as a potential risk factor following analyses using the Cockcroft–Gault equation; however, a subsequent post hoc analysis using the CKD-EPI equation did not demonstrate a clear association between renal impairment severity and ILD/pneumonitis risk [5]. Taken together, these observations suggest that clinical risk factors identified in larger datasets may not be uniformly detected across smaller or more homogeneous cohorts.

Notably, HER2 expression assessed by IHC was not associated with ILD risk in this study, which is consistent with the mechanistic and clinical evidence indicating that pulmonary toxicity related to HER2-directed antibody–drug conjugates does not correlate with target antigen expression levels in lung tissue. Taken together, although clinical characteristics remain essential for contextualizing patient risk and informing clinical vigilance, their ability to discriminate ILD susceptibility in individual patients appears to be limited in the context of T-DXd therapy.

These considerations motivated further investigation into host-related factors beyond conventional clinical parameters, including genetic susceptibility, which may contribute to inter-individual variability in pulmonary toxicity.

Using a genome-wide approach with genotype imputation, we did not identify any SNPs that were statistically significant after correcting for multiple tests. This finding is consistent with a previous observation that drug-induced ILD is unlikely to be driven by a single high-penetrance genetic variant [16]. In GWAS, stringent correction for multiple comparisons, such as Bonferroni adjustment, substantially reduces statistical power, particularly in studies with limited sample sizes, and may lead to underestimation of true associations with modest effect sizes.

In contrast, when we focused on SNPs previously reported to be associated with ILD in a Japanese population treated with other anticancer agents, one variant demonstrated a statistically significant association in our cohort. This candidate-based approach reduces the burden of multiple testing and may be better suited for detecting modest genetic effects in relatively small cohorts.

Notably, the SNP identified in our study showed an association in the opposite direction to that previously reported in which the variant was described as a risk allele for ILD [16]. In our cohort, the same variant was more frequent in patients who did not develop ILD.

The previous study analyzed 44 cases of drug-induced ILD in Japanese patients with various cancer types, including 7 breast cancer cases, and compared them with both general population controls and chemotherapy-tolerant controls. The implicated drugs included gefitinib, erlotinib, nivolumab, trastuzumab (±paclitaxel), and gemcitabine (±nab-paclitaxel). In the study, rs12625311 demonstrated a significant association with drug-induced ILD when all cases were analyzed together. However, subgroup analyses by breast cancer did not show significant associations and subgroup analysis restricted to anti-HER2 therapies was not performed [16].

Several possible explanations may account for the discrepant direction of association observed in our study: the functional impact of the SNP may depend on the context and may be influenced by differences in drug mechanisms, cancer type, or patient characteristics. In addition, treatment-specific immune activation or toxicity pathways may modify genetic effects. Furthermore, differences in study design and control selection may have an influence. Moreover, differences in linkage dis-equilibrium structure across cohorts may have resulted in the observed variants tagging different causal loci. Alternatively, the variant may exert protective effects under certain biological conditions.

These findings highlight the importance of the cautious interpretation of genetic associations in drug-induced toxicities and emphasize the need for validation in independent cohorts treated with the same agent.

The SNP identified in this study was located within the intronic region of *TSHZ2*. To date, no study has directly linked *TSHZ2* to ILD or pulmonary toxicity. *TSHZ2* has been characterized as a transcriptional regulator with tumor suppressor functions, including cell cycle regulation and tumor progression suppression [27]. Experimental studies have shown that *TSHZ2* expression is regulated downstream of epidermal growth factor receptor signaling and participates in the complexes involved in cytokinesis and cellular proliferation. In addition, genetic variants in *TSHZ2* have previously been implicated in susceptibility to Stevens–Johnson syndrome/toxic epidermal necrolysis with severe ocular complications in Japanese individuals [28]. As these conditions represent severe immune-mediated adverse reactions, this association raises the possibility that *TSHZ2* may participate in immune regulatory pathways. Emerging transcriptomic and epigenetic analyses have identified *TSHZ2* expression in naïve CD4 + T cells, in which it appears to influence cellular longevity and proliferative capacity [29]. These findings suggest a potential role in immune regulatory processes. Drug-induced ILD is thought to involve dysregulated immune and inflammatory responses in susceptible individuals. Although no direct evidence links *TSHZ2* to pulmonary toxicity, intronic variants may influence gene expression. It is therefore conceivable that genetic variation in *TSHZ2* could contribute to interindividual differences in drug response. However, no functional studies of rs12625311 have been reported to data, and the biological mechanism underlying this association remains uncertain.

This study has several limitations. First, the relatively small sample size limited the statistical power, particularly in detecting modest associations between clinical factors or genetic variants and ILD development. In addition, the relative clinical homogeneity of the cohort may have further reduced variability in baseline risk factors. Furthermore, because all participants in this study were Japanese and treated at a single institution, caution is warranted when extrapolating these findings to other populations. Therefore, the absence of statistically significant clinical predictors in this cohort should be interpreted with caution and does not exclude the potential contribution of such factors in larger or more heterogeneous populations, as suggested in previous studies. Given these constraints, this study should be considered an exploratory pilot analysis. Larger, multi-center cohorts are required to validate these findings and to enable adequately powered analyses, particularly for genetic association studies. Second, as this was a retrospective study, the design may have limited the uniformity of clinical assessments, and the possibility of selection bias cannot be excluded. Third, although genotype imputation has expanded genomic coverage, rare and structural variants may not have been adequately captured. Fourth, functional validation of the identified SNP was not performed, precluding definitive conclusions regarding associated biological mechanisms.

Despite these limitations, the integrated evaluation of clinical and genetic factors provides complementary insights into the complex and multifactorial nature of T-DXd-induced ILD. Given the absence of established predictive biomarkers for T-DXd-induced ILD, identification of potential host susceptibility factors may help identify patients at increased risk before treatment initiation. If validated in larger, prospectively designed studies, the variant could inform risk-adapted monitoring and more individualized treatment decisions, potentially improving patient safety.

## 5. Conclusions

In the present study, no baseline clinical or treatment-related factors were significantly associated with T-DXd-induced ILD. Although genome-wide analyses did not identify high-penetrance variants, a targeted candidate-based genetic approach suggested the potential contribution of host genetic variation to ILD susceptibility. Collectively, these findings support the multifactorial pathogenesis of T-DXd-induced ILD, warranting further investigation in a larger prospective cohort to clarify the clinical utility of genetic risk stratification.

## Figures and Tables

**Table 1:** Baseline characteristics of patients.

Baseline Characteristics	ILD+ (*n* = 15)	ILD− (*n* = 39)
**Age**		
Median ± SD (range), years	60.9 ± 11.6 (39–81)	56.6 ± 11.9 (35–82)
<65 years, *n* (%)	9 (60.0%)	28 (71.8%)
≥65 years, *n* (%)	6 (40.0%)	11 (28.2%)
**Sex**, *n* (%)		
Female	15 (100%)	39 (100%)
**ECOG performance status**, *n* (%)		
0	10 (66.7%)	30 (76.9%)
≥1	5 (33.3%)	9 (23.1%)
**Lung comorbidities before/during T-DXd treatment** **(history of ILD or pneumonitis),** *n* (%)		
Yes	2 (13.3%)	5 (12.8%)
**History of smoking**, *n* (%)		
Yes	3 (20.0%)	5 (12.8%)
missing	2 (13.3%)	3 (7.7%)
**Lung metastasis**	1 (6.7%)	1 (2.6%)
**Pleura metastasis**	0 (0%)	2 (5.1%)
**Baseline renal function (CrCl)**		
Median ± SD (range), mL/min	77.8 ± 28.7 (46.4–139.9)	86.1 ± 26.5 (40.9–162.4)
CrCl ≥ 90 mL/min, *n* (%)	3 (20.0%)	17 (43.6%)
CrCl < 90 mL/min, *n* (%)	12 (80.0%)	22 (56.4%)
**Body weight**, median ± SD (range), kg	50.6 ± 11.5 (40.5–72.5)	53.3 ± 12.6 (31.6–80.0)
Baseline T-DXd dose, *n* (%)		
≤5.4 mg/kg	13 (86.7%)	37 (94.9%)
≥6.4 mg/kg	2 (13.3%)	2 (5.1%)
**Prior lines of therapy in the metastatic setting**		
≤3	11 (73.3%)	20 (51.3%)
≥4	4 (26.7%)	19 (48.7%)
**Estrogen Receptor**, *n* (%)		
+	10 (66.7%)	28 (71.8%)
−	5 (33.3%)	11 (28.2%)
**HER2**, *n* (%)		
+	8 (53.3%)	13 (33.3%)
−	7 (46.7%)	26 (66.7%)
**HER2 IHC score**, *n* (%)		
1+	5 (33.3%)	16 (41.0%)
2+	3 (20.0%)	14 (35.9%)
3+	7 (46.7%)	9 (23.1%)

**Table 2:** Analyses of factors associated with ILD development.

Variable	OR (95% CI)	*p* Value
Age ≥65 vs. <65 years	1.70 (0.50–5.70)	0.39
ECOG PS ≥1 vs. 0	1.68 (0.48–5.95)	0.42
Lung comorbidity: Yes vs. No	1.16 (0.23–5.89)	0.86
Smoking history: Yes vs. No	1.76 (0.41–7.57)	0.45
Lung or Pleura metastasis: Yes vs. No	1.08 (0.15–7.95)	0.94
CrCl <90 vs. ≥90 mL/min	2.78 (0.72–10.68)	0.14
Baseline dose ≥6.4 vs. ≤5.4 mg/kg	2.78 (0.44–17.57)	0.28
Prior lines of therapy ≥4 vs. ≤3	0.41 (0.12–1.45)	0.17
ER-positive vs. ER-negative	0.77 (0.22–2.68)	0.68
HER2-positive vs. HER2-negative	2.22 (0.68–7.26)	0.19
HER2 IHC ≥2+ vs. ≤1+	1.34 (0.40–4.49)	0.63

**Table 3:** Case–control study of SNPs associated with T-DXd-induced ILD.

Chr	SNP ID	Position	Allele (1/2)	Risk Allele * [16]	ILD					Non-ILD					*p* Value	OR (95% Cl)
					Allele		Allele Frequency		RAF	Allele		Allele Frequency		RAF		
					1	2	1	2		1	2	1	2			
20	rs12625311	51,836,793	T/C	C	25	5	0.83	0.17	0.17	43	35	0.55	0.45	0.45	1.59 × 10^−2^	0.24
																[0.07–0.77]

Chr: chromosome. RAF: risk allele frequency. The reference allele (GRCh37) was defined as allele 1. * The direction of the effect was reversed from that reported previously [16].

## Data Availability

Data is unavailable due to privacy or ethical restrictions.

## Abbreviations

The following abbreviations are used in this manuscript:

T-DXd	Trastuzumab deruxtecan
HER2	Human epidermal growth factor receptor 2
ILD	Interstitial lung disease
IHC	Immunohistochemistry
SNPs	Single-nucleotide polymorphisms
CT	Computed tomography
CTCAE	Common Terminology Criteria for Adverse Events
GWAS	Genome-wide association study
CrCl	Creatinine clearance
ORs	Odds ratios
CIs	Confidence intervals
QC	Quality control
MAF	Minor allele frequency
ECOG	Eastern Clinical Oncology Group
IQRs	Interquartile ranges
Chr	Chromosome
RAF	Risk allele frequency
